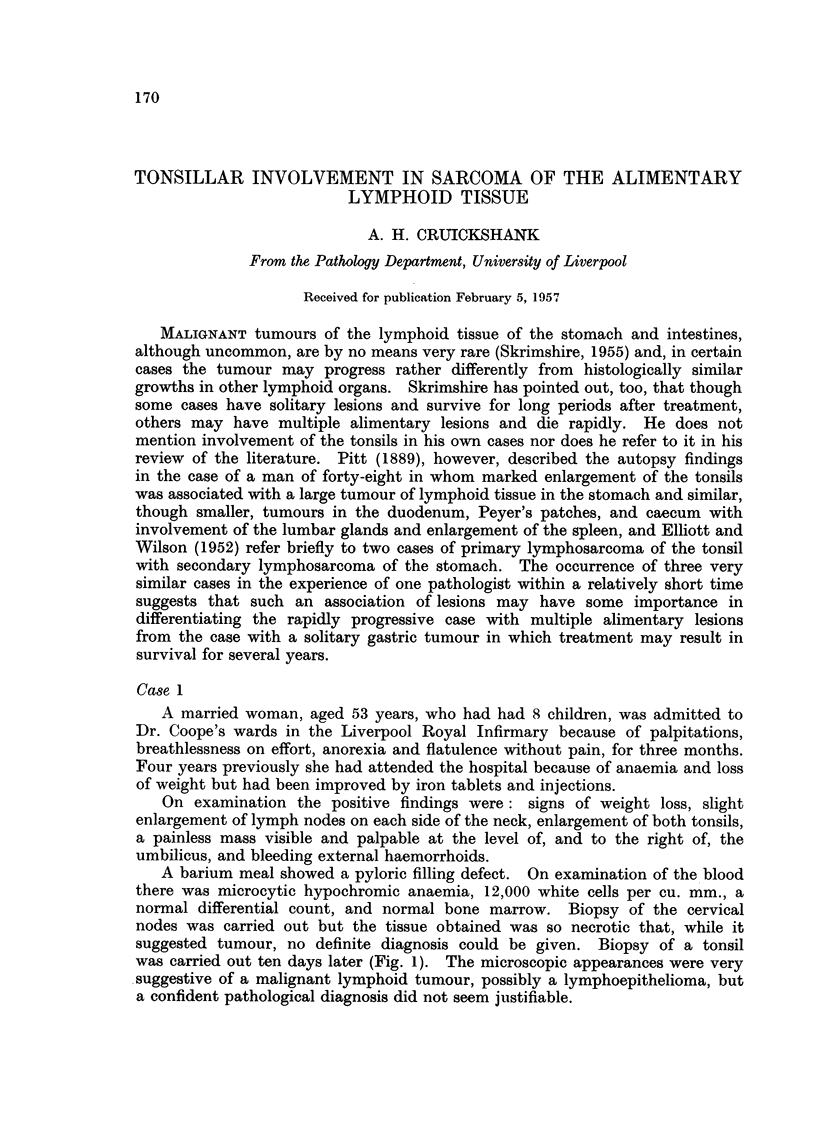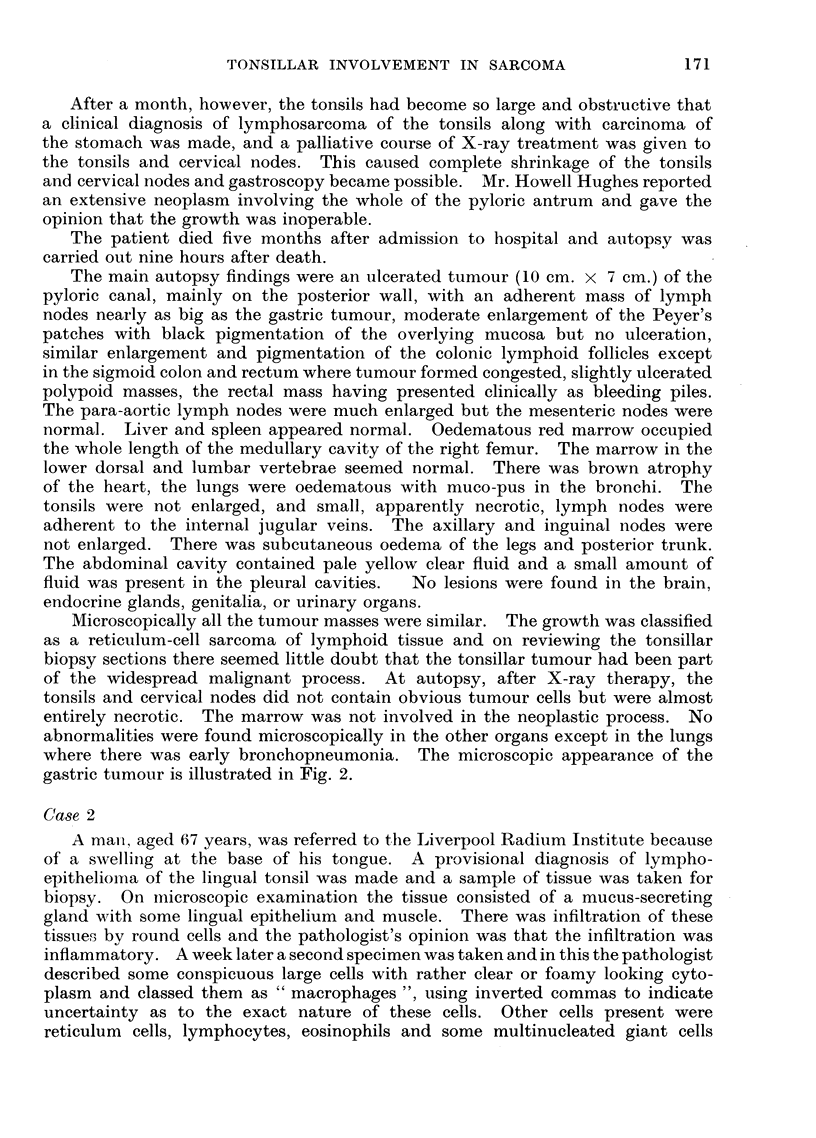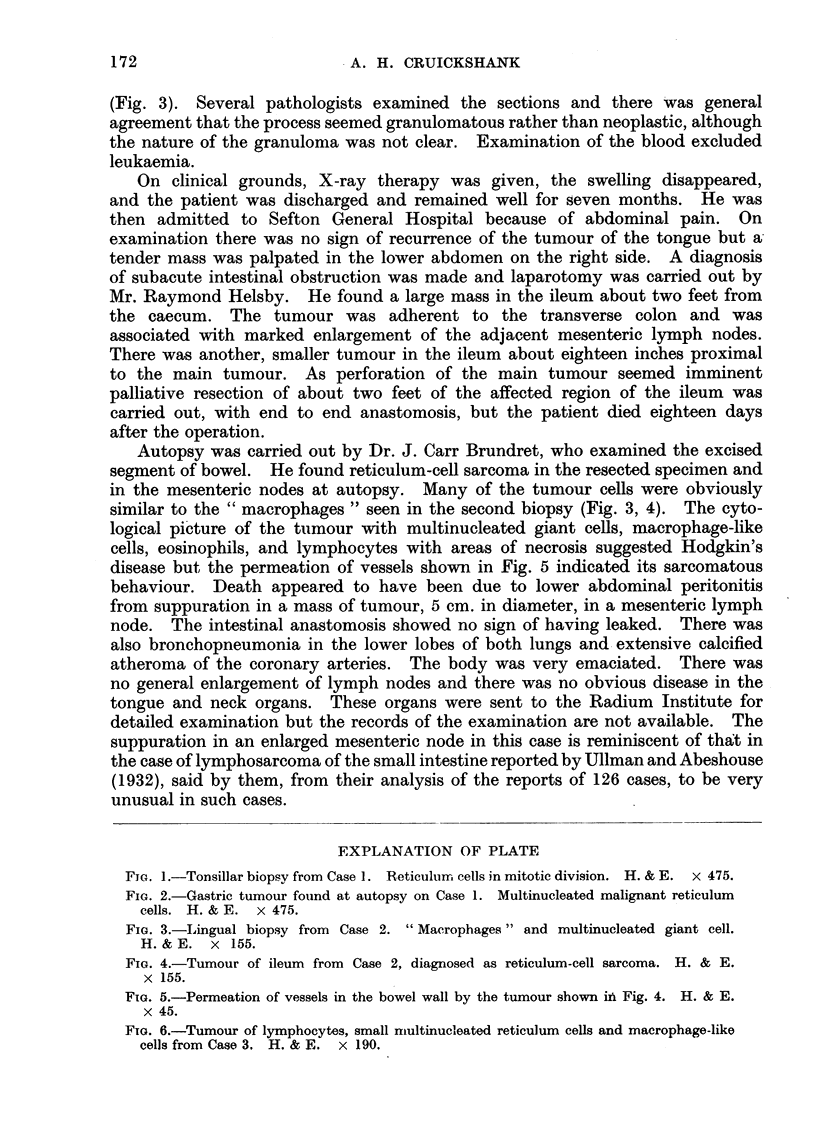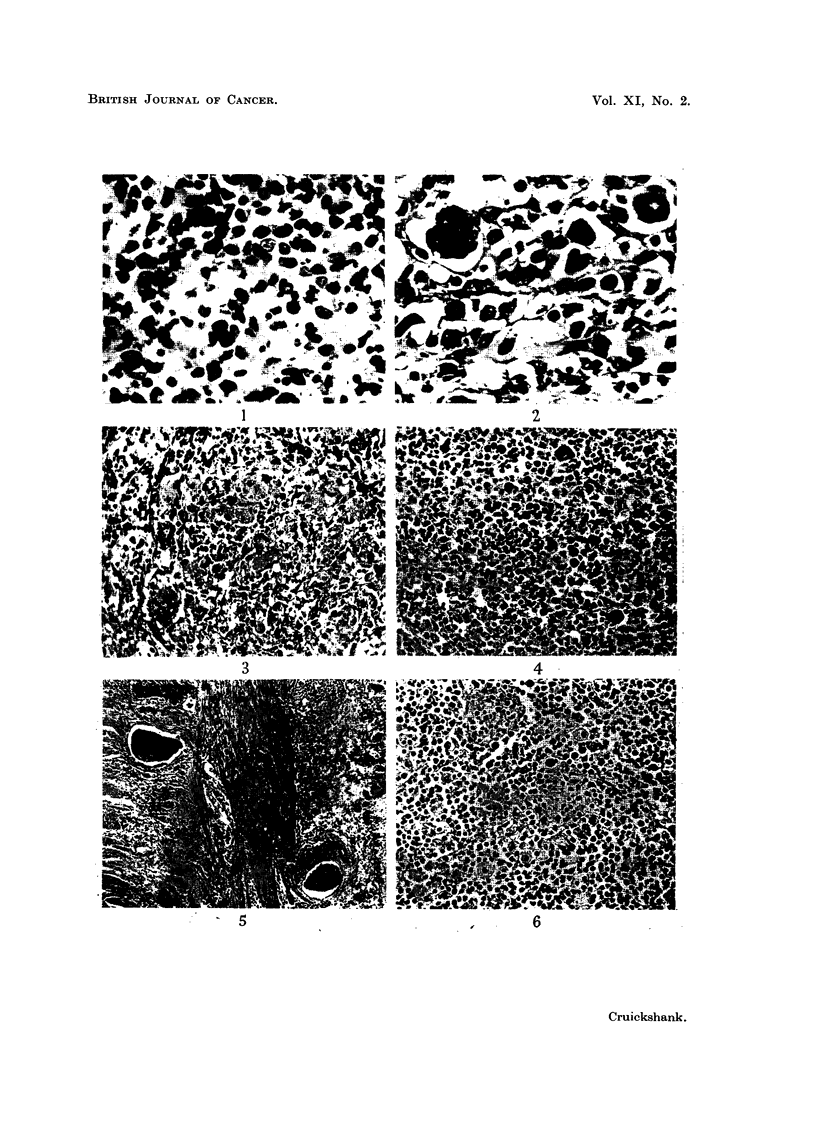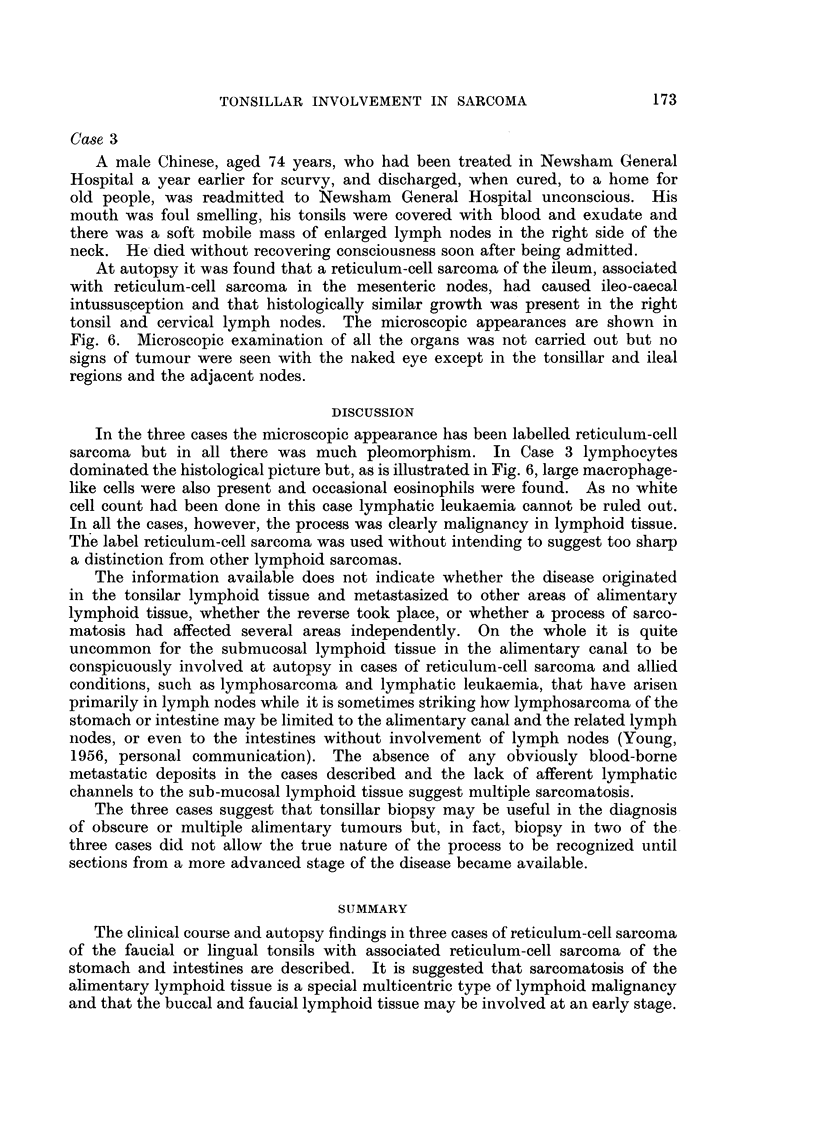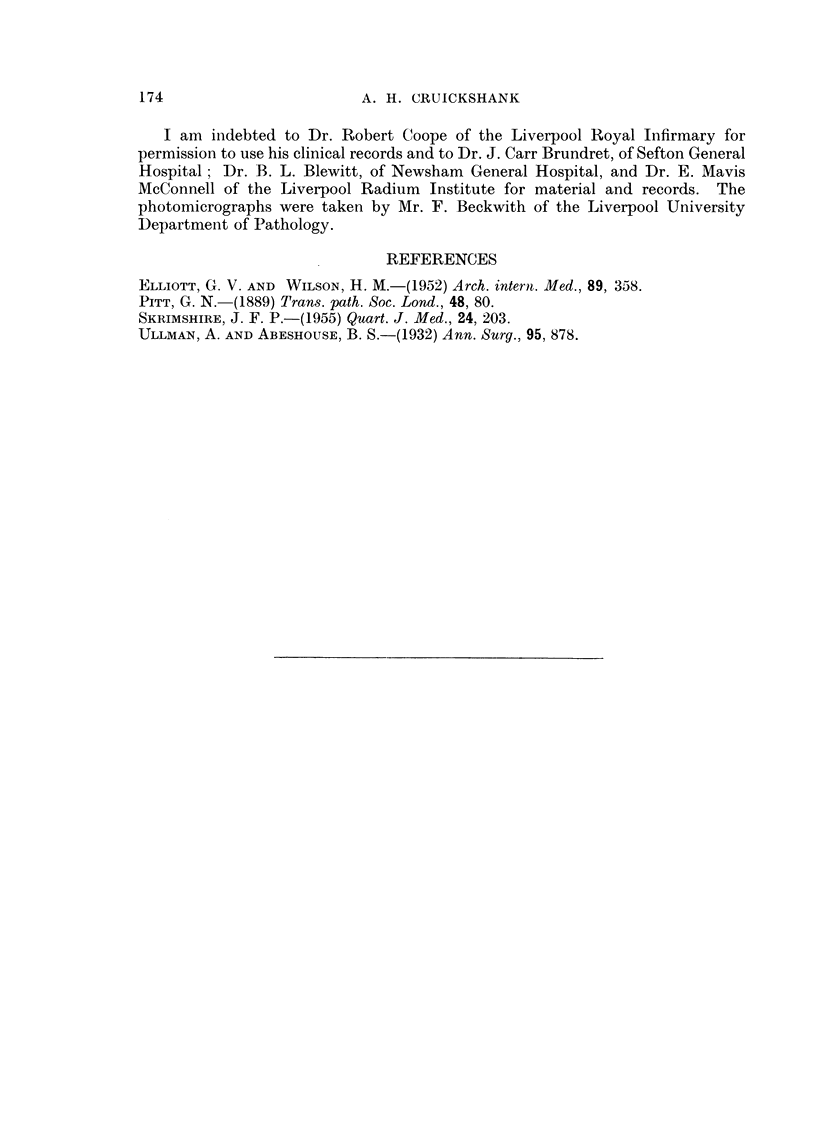# Tonsillar Involvement in Sarcoma of the Alimentary Lymphoid Tissue

**DOI:** 10.1038/bjc.1957.23

**Published:** 1957-06

**Authors:** A. H. Cruickshank

## Abstract

**Images:**


### TONSILLAR INVOLVEMENT IN SARCOMA OF THE ALIMENTARY LYMPHOID TISSUE

A. H. CRUICKSHANK

*From the Pathology Department, University of Liverpool*

Received for publication February 5, 1957

MALIGNANT tumours of the lymphoid tissue of the stomach and intestines, although uncommon, are by no means very rare (Skrimshire, 1955) and, in certain cases the tumour may progress rather differently from histologically similar growths in other lymphoid organs. Skrimshire has pointed out, too, that though some cases have solitary lesions and survive for long periods after treatment, others may have multiple alimentary lesions and die rapidly. He does not mention involvement of the tonsils in his own cases nor does he refer to it in his review of the literature. Pitt (1889), however, described the autopsy findings in the case of a man of forty-eight in whom marked enlargement of the tonsils was associated with a large tumour of lymphoid tissue in the stomach and similar, though smaller, tumours in the duodenum, Peyer's patches, and caecum with involvement of the lumbar glands and enlargement of the spleen, and Elliott and Wilson (1952) refer briefly to two cases of primary lymphosarcoma of the tonsil with secondary lymphosarcoma of the stomach. The occurrence of three very similar cases in the experience of one pathologist within a relatively short time suggests that such an association of lesions may have some importance in differentiating the rapidly progressive case with multiple alimentary lesions from the case with a solitary gastric tumour in which treatment may result in survival for several years.

*Case* 1

A married woman, aged 53 years, who had had 8 children, was admitted to Dr. Coope's wards in the Liverpool Royal Infirmary because of palpitations, breathlessness on effort, anorexia and flatulence without pain, for three months. Four years previously she had attended the hospital because of anaemia and loss of weight but had been improved by iron tablets and injections.

On examination the positive findings were: signs of weight loss, slight enlargement of lymph nodes on each side of the neck, enlargement of both tonsils, a painless mass visible and palpable at the level of, and to the right of, the umbilicus, and bleeding external haemorrhoids.

A barium meal showed a pyloric filling defect. On examination of the blood there was microcytic hypochromic anaemia, 12,000 white cells per cu. mm., a normal differential count, and normal bone marrow. Biopsy of the cervical nodes was carried out but the tissue obtained was so necrotic that, while it suggested tumour, no definite diagnosis could be given. Biopsy of a tonsil was carried out ten days later (Fig. 1). The microscopic appearances were very suggestive of a malignant lymphoid tumour, possibly a lymphoepithelioma, but a confident pathological diagnosis did not seem justifiable.

After a month, however, the tonsils had become so large and obstructive that a clinical diagnosis of lymphosarcoma of the tonsils along with carcinoma of the stomach was made, and a palliative course of X-ray treatment was given to the tonsils and cervical nodes. This caused complete shrinkage of the tonsils and cervical nodes and gastroscopy became possible. Mr. Howell Hughes reported an extensive neoplasm involving the whole of the pyloric antrum and gave the opinion that the growth was inoperable.

The patient died five months after admission to hospital and autopsy was carried out nine hours after death.

The main autopsy findings were an ulcerated tumour (10 cm. × 7 cm.) of the pyloric canal, mainly on the posterior wall, with an adherent mass of lymph nodes nearly as big as the gastric tumour, moderate enlargement of the Peyer's patches with black pigmentation of the overlying mucosa but no ulceration, similar enlargement and pigmentation of the colonic lymphoid follicles except in the sigmoid colon and rectum where tumour formed congested, slightly ulcerated polypoid masses, the rectal mass having presented clinically as bleeding piles. The para-aortic lymph nodes were much enlarged but the mesenteric nodes were normal. Liver and spleen appeared normal. Oedematous red marrow occupied the whole length of the medullary cavity of the right femur. The marrow in the lower dorsal and lumbar vertebrae seemed normal. There was brown atrophy of the heart, the lungs were oedematous with muco-pus in the bronchi. The tonsils were not enlarged, and small, apparently necrotic, lymph nodes were adherent to the internal jugular veins. The axillary and inguinal nodes were not enlarged. There was subcutaneous oedema of the legs and posterior trunk. The abdominal cavity contained pale yellow clear fluid and a small amount of fluid was present in the pleural cavities. No lesions were found in the brain, endocrine glands, genitalia, or urinary organs.

Microscopically all the tumour masses were similar. The growth was classified as a reticulum-cell sarcoma of lymphoid tissue and on reviewing the tonsillar biopsy sections there seemed little doubt that the tonsillar tumour had been part of the widespread malignant process. At autopsy, after X-ray therapy, the tonsils and cervical nodes did not contain obvious tumour cells but were almost entirely necrotic. The marrow was not involved in the neoplastic process. No abnormalities were found microscopically in the other organs except in the lungs where there was early bronchopneumonia. The microscopic appearance of the gastric tumour is illustrated in Fig. 2.

*Case* 2

A man, aged 67 years, was referred to the Liverpool Radium Institute because of a swelling at the base of his tongue. A provisional diagnosis of lympho-epithelioma of the lingual tonsil was made and a sample of tissue was taken for biopsy. On microscopic examination the tissue consisted of a mucus-secreting gland with some lingual epithelium and muscle. There was infiltration of these tissues by round cells and the pathologist's opinion was that the infiltration was inflammatory. A week later a second specimen was taken and in this the pathologist described some conspicuous large cells with rather clear or foamy looking cyto-plasm and classed them as " macrophages ", using inverted commas to indicate uncertainty as to the exact nature of these cells. Other cells present were reticulum cells, lymphocytes, eosinophils and some multinucleated giant cells

(Fig. 3). Several pathologists examined the sections and there was general agreement that the process seemed granulomatous rather than neoplastic, although the nature of the granuloma was not clear. Examination of the blood excluded leukaemia.

On clinical grounds, X-ray therapy was given, the swelling disappeared, and the patient was discharged and remained well for seven months. He was then admitted to Sefton General Hospital because of abdominal pain. On examination there was no sign of recurrence of the tumour of the tongue but a tender mass was palpated in the lower abdomen on the right side. A diagnosis of subacute intestinal obstruction was made and laparotomy was carried out by Mr. Raymond Helsby. He found a large mass in the ileum about two feet from the caecum. The tumour was adherent to the transverse colon and was associated with marked enlargement of the adjacent mesenteric lymph nodes. There was another, smaller tumour in the ileum about eighteen inches proximal to the main tumour. As perforation of the main tumour seemed imminent palliative resection of about two feet of the affected region of the ileum was carried out, with end to end anastomosis, but the patient died eighteen days after the operation.

Autopsy was carried out by Dr. J. Carr Brundret, who examined the excised segment of bowel. He found reticulum-cell sarcoma in the resected specimen and in the mesenteric nodes at autopsy. Many of the tumour cells were obviously similar to the " macrophages " seen in the second biopsy (Fig. 3, 4). The cytological picture of the tumour with multinucleated giant cells, macrophage-like cells, eosinophils, and lymphocytes with areas of necrosis suggested Hodgkin's disease but the permeation of vessels shown in Fig. 5 indicated its sarcomatous behaviour. Death appeared to have been due to lower abdominal peritonitis from suppuration in a mass of tumour, 5 cm. in diameter, in a mesenteric lymph node. The intestinal anastomosis showed no sign of having leaked. There was also bronchopneumonia in the lower lobes of both lungs and extensive calcified atheroma of the coronary arteries. The body was very emaciated. There was no general enlargement of lymph nodes and there was no obvious disease in the tongue and neck organs. These organs were sent to the Radium Institute for detailed examination but the records of the examination are not available. The suppuration in an enlarged mesenteric node in this case is reminiscent of that in the case of lymphosarcoma of the small intestine reported by Ullman and Abeshouse (1932), said by them, from their analysis of the reports of 126 cases, to be very unusual in such cases.

---

EXPLANATION OF PLATE

FIG. 1.—Tonsillar biopsy from Case 1. Reticulum cells in mitotic division. H. & E. × 475.

FIG. 2.—Gastric tumour found at autopsy on Case 1. Multinucleated malignant reticulum cells. H. & E. × 475.

FIG. 3.—Lingual biopsy from Case 2. " Macrophages " and multinucleated giant cell. H. & E. × 155.

FIG. 4.—Tumour of ileum from Case 2, diagnosed as reticulum-cell sarcoma. H. & E. × 155.

FIG. 5.—Permeation of vessels in the bowel wall by the tumour shown in Fig. 4. H. & E. × 45.

FIG. 6.—Tumour of lymphocytes, small multinucleated reticulum cells and macrophage-like cells from Case 3. H. & E. × 190.

1

2

3

4

5

6

Cruickshank.

*Case* 3

A male Chinese, aged 74 years, who had been treated in Newsham General Hospital a year earlier for scurvy, and discharged, when cured, to a home for old people, was readmitted to Newsham General Hospital unconscious. His mouth was foul smelling, his tonsils were covered with blood and exudate and there was a soft mobile mass of enlarged lymph nodes in the right side of the neck. He died without recovering consciousness soon after being admitted.

At autopsy it was found that a reticulum-cell sarcoma of the ileum, associated with reticulum-cell sarcoma in the mesenteric nodes, had caused ileo-caecal intussusception and that histologically similar growth was present in the right tonsil and cervical lymph nodes. The microscopic appearances are shown in Fig. 6. Microscopic examination of all the organs was not carried out but no signs of tumour were seen with the naked eye except in the tonsillar and ileal regions and the adjacent nodes.

### DISCUSSION

In the three cases the microscopic appearance has been labelled reticulum-cell sarcoma but in all there was much pleomorphism. In Case 3 lymphocytes dominated the histological picture but, as is illustrated in Fig. 6, large macrophage-like cells were also present and occasional eosinophils were found. As no white cell count had been done in this case lymphatic leukaemia cannot be ruled out. In all the cases, however, the process was clearly malignancy in lymphoid tissue. The label reticulum-cell sarcoma was used without intending to suggest too sharp a distinction from other lymphoid sarcomas.

The information available does not indicate whether the disease originated in the tonsilar lymphoid tissue and metastasized to other areas of alimentary lymphoid tissue, whether the reverse took place, or whether a process of sarcomatosis had affected several areas independently. On the whole it is quite uncommon for the submucosal lymphoid tissue in the alimentary canal to be conspicuously involved at autopsy in cases of reticulum-cell sarcoma and allied conditions, such as lymphosarcoma and lymphatic leukaemia, that have arisen primarily in lymph nodes while it is sometimes striking how lymphosarcoma of the stomach or intestine may be limited to the alimentary canal and the related lymph nodes, or even to the intestines without involvement of lymph nodes (Young, 1956, personal communication). The absence of any obviously blood-borne metastatic deposits in the cases described and the lack of afferent lymphatic channels to the sub-mucosal lymphoid tissue suggest multiple sarcomatosis.

The three cases suggest that tonsillar biopsy may be useful in the diagnosis of obscure or multiple alimentary tumours but, in fact, biopsy in two of the three cases did not allow the true nature of the process to be recognized until sections from a more advanced stage of the disease became available.

### SUMMARY

The clinical course and autopsy findings in three cases of reticulum-cell sarcoma of the faucial or lingual tonsils with associated reticulum-cell sarcoma of the stomach and intestines are described. It is suggested that sarcomatosis of the alimentary lymphoid tissue is a special multicentric type of lymphoid malignancy and that the buccal and faucial lymphoid tissue may be involved at an early stage.

I am indebted to Dr. Robert Coope of the Liverpool Royal Infirmary for permission to use his clinical records and to Dr. J. Carr Brundret, of Sefton General Hospital; Dr. B. L. Blewitt, of Newsham General Hospital, and Dr. E. Mavis McConnell of the Liverpool Radium Institute for material and records. The photomicrographs were taken by Mr. F. Beckwith of the Liverpool University Department of Pathology.

### REFERENCES

Elliott, G. V. and Wilson, H. M.—(1952) *Arch. intern. Med.*, **89**, 358.
Pitt, G. N.—(1889) *Trans. path. Soc. Lond.*, **48**, 80.
Skrimshire, J. F. P.—(1955) *Quart. J. Med.*, **24**, 203.
Ullman, A. and Abeshouse, B. S.—(1932) *Ann. Surg.*, **95**, 878.